# Pilot Implementation of an Intervention Program to Promote Fine Motor Skills in Preschoolers: Feasibility and Acceptability

**DOI:** 10.1155/oti/9903335

**Published:** 2026-02-24

**Authors:** Suchitporn Lersilp, Kewalin Panyo, Napalai Chaimaha, Supawadee Putthinoi, Autchariya Punyakaew, Sasithorn Sung-U

**Affiliations:** ^1^ Department of Occupational Therapy, Faculty of Associated Medical Sciences, Chiang Mai University, Chiang Mai, Thailand, cmu.ac.th

**Keywords:** acceptability, feasibility, fine motor, intervention program, preschooler

## Abstract

**Background:**

Early intervention programs are needed for children at risk of fine motor delays. Due to the scarcity of community occupational therapists, alternative services are being developed through collaboration with related professionals.

**Objective:**

This study was aimed at evaluating the feasibility and acceptability of a pilot intervention program designed to promote fine motor skill development in preschoolers attending early childhood educational settings in a community.

**Methods:**

A pilot quasi‐experimental design was employed. Thirty preschoolers (aged 2–6 years) were identified as at‐risk for fine motor delays and recruited from early childhood educational settings in a community in Chiang Mai province, Thailand. The intervention program was tailored to five age groups and comprised 20 activity sessions (10 h total) that focused on both bilateral motor coordination and visual–motor integration. Content validity of the program was assessed by five experts. Feasibility was evaluated by using McNemar’s test. Acceptability was explored through a focus group of seven teachers as program users. Then, qualitative data were analyzed by thematic analysis.

**Results:**

McNemar’s test was employed to evaluate the change in fine motor development for paired dichotomous data (“still delayed” vs. “normal/improved”). At postintervention, 22 out of 30 children (73.33%) demonstrated improved fine motor development, with significant statistical support (*χ* ^2^ = 5.63, *p* < 0.001, *α* = 0.05). Odds ratio of improvement was 2.75, with 95% confidence interval (CI) (95% CI: 1.22–6.18), indicating the magnitude of the effect. The highest developmental gains were observed in children aged 4.0–4.11 years (Group III). Thematic analysis revealed three core themes indicating acceptability: (1) engagement and appropriateness of materials, (2) effectiveness in promoting fine motor and academic skills, and (3) practicality and usability for teachers.

**Conclusion:**

This teacher‐led collaborative service provides promising preliminary evidence of a feasible and acceptable approach to promoting fine motor skills in at‐risk preschoolers, though further rigorous studies are needed to confirm its efficacy.

## 1. Introduction

The preschooler period is the stage of early childhood. Its advancement covers various domains of developmental milestones including cognitive, social–emotional, physical, and language development, which are crucial for preparing children for future educational experiences. Experience from play and age‐appropriated activity during early childhood can become physiologically imprinted and impact outcomes throughout the child’s life [1]. Learning experience is important for their growth as well as encouragement in building trusting relationships, enhancing communication skills, and enabling executive function, particularly in planning, problem solving, and decision‐making [2].

Occupational therapists focus on creating or facilitating opportunities for preschoolers to engage in age‐appropriated occupations that lead to their participation in activities [3]. Fine motor skills are observable, goal‐directed actions that result in the quality of occupational performance [3]. Effective use of motor performance skills is demonstrated when the client carries out activities efficiently, safely, and with ease or without assistance. Conversely, ineffective use of performance skills demonstrates the need for assistance or support in performing their activities. Thus, fine motor skills in preschoolers are critical in the coordination of hand and finger movements. During early childhood, children refine fine motor skills to perform tasks such as drawing, cutting, and manipulating small objects, which support daily activities and academic readiness. When supporting preschoolers at risk of delays in fine motor development, it is important to provide early intervention with ample opportunities to practice through engagement in various manipulative activities that enhance dexterity, eye–hand coordination, and spatial concepts [4]. These early interventions not only support the child’s acquisition of necessary academic skills but also build self‐esteem and confidence in their abilities, which ultimately prepare them for successful transition into formal education [5–9].

Interestingly, national health surveys indicated that approximately 25% of children in low‐ and middle‐income countries were suspected of having developmental delays [10]. This shows the various challenges faced by children globally. Thailand has encountered similar challenges, with developmental delays among preschoolers remaining a significant concern. Various factors contribute to delayed development among preschoolers, such as maternal education levels, socioeconomic status, environmental influences, and less opportunity to access developmental stimulation services. Therefore, tangible policies and health strategies are necessary to encourage effective early intervention that mitigates the risks associated with these delays [11, 12]. However, in real community contexts, there has been a lack of occupational therapists to provide services for preschoolers with delayed development, despite the government’s policy to promote progression in early childhood. Moreover, the policy stresses that preschool teachers in the community have to screen their students’ development and receive referrals based on a developmental screening tool: the Developmental Surveillance and Promotion Manual (DSPM), from local healthcare service providers. However, when developmental delays are identified, teachers often experience a lack of confidence in providing appropriate classroom support.

A scarcity of professionals specializing in developmental promotion in the community, particularly occupational therapists, can limit children’s access to timely and age‐appropriate developmental stimulation [13]. By prioritizing the development of fine motor skills through well‐designed early intervention programs, collaboration between teachers and occupational therapists should be emphasized to reduce direct service limitations in the community. Moreover, this collaborative work can enhance the teachers’ confidence in promoting fine motor skills in their students within the community, while preschoolers benefit from fine motor skill development without delayed referrals, intensity, and insufficient service frequency.

Despite the recognized clinical and academic importance of early fine motor skill development, a significant gap remains in the practical implementation within resource‐limited community settings. Current practice often relies on professional specialists and tends to focus on isolated subcomponents, with limited evaluation of teacher‐led implementation in resource‐constrained community settings [14]. Consequently, occupational therapy research, which systematically evaluates teacher‐led collaborative intervention programs, is notably scarce, particularly when employing a structured dual‐component focus and age‐progressive sequence that aligns with developmental milestones within routine preschool workflows. This gap necessitates this study, which was aimed at exploring the feasibility and acceptability of a pilot intervention program designed to promote fine motor skill development for preschoolers attending community educational settings.

The study addressed two primary research questions:

1. Will the intervention improve preschool students with suspected fine motor delays?
2. Will the teachers, as program users, be satisfied with the implemented intervention program?

Assessing the feasibility of the pilot intervention was crucial in determining its practical viability, implementation fidelity, and potential effectiveness within existing early childhood educational structures. Concurrently, it was paramount to evaluate the teachers’ acceptability, as primary program users, to ensure that the program is perceived as practical, usable, and engaging. This strengthened the likelihood of successful integration and long‐term sustainability as a resource to enhance age‐appropriate fine motor development in at‐risk preschoolers. For these reasons, the expected benefit of this study was the emergence of an intervention program that would act as promising preliminary evidence of not only enhancing age‐appropriate preschooler development but also the teachers’ confidence in providing classroom activities for students with developmental delay.

## 2. Materials and Methods

This research had a pilot quasi‐experimental design, aimed at implementing an intervention program that promotes fine motor skill development in preschoolers in a community. This design focused on assessing the feasibility and acceptability of the intervention. However, because of its one‐group pretest/posttest design, without a control or comparison group, the ability to infer a strong cause‐and‐effect relationship between intervention and fine motor improvement was limited.

### 2.1. Participants

The participants were preschoolers, aged 2–6 years. They were currently enrolled for the 2024 academic year in early childhood development centers in a community in Chiang Mai province, Thailand, and monitored for developmental delays in fine motor skills by using the DSPM screening tool. The samples were determined through quota sampling based on gender and age groups to ensure representation of the participants. Finally, 30 participants were recruited to this pilot study. The mean age at the program entry was 53.87 months (SD = 16.40). The maximum and minimum ages were 82 and 28 months, respectively. They included 16 (53.33%) male and 14 (46.67%) female preschoolers. Most of them (23.33%) were in Age Group III (4.0–4.11 years). In addition, most (40.00%) were studying in Kindergarten 2. Demographics of the participants are presented in Table 1.

**Table 1:** Demographic characteristics of the participants (*n* = 30).

Characteristics	Number (%)
*Gender*	
Male	16 (53.33)
Female	14 (46.67)
*Age group*	
Age Group I (2.0–2.11 years)	5 (16.67)
Age Group II (3.0–3.11 years)	6 (20.00)
Age Group III (4.0–4.11 years)	7 (23.33)
Age Group IV (5.0–5.11 years)	6 (20.00)
Age Group V (6.0–6.11 years)	6 (20.00)
*Education*	
Prekindergarten	6 (20.00)
Kindergarten 1	6 (20.00)
Kindergarten 2	12 (40.00)
Kindergarten 3	6 (20.00)

### 2.2. Data Collection

There were two phases of this study. Phase 1 developed an intervention program to promote fine motor skills in preschoolers. The activities of the program were designed by analyzing and reviewing relevant literature. Therefore, they were categorized according to two fundamental skill components: bilateral motor coordination and visual–motor integration. This was structured across five age groups: Group I: 2.0–2.11 years; Group II: 3.0–3.11 years; Group III: 4.0–4.11 years; Group IV: 5.0–5.11 years; and Group V: 6.0–6.11 years. Content validity was examined by five experts, including one pediatric occupational therapist, one academic occupational therapist, one occupational therapist with at least 3 years’ experience in promoting fine motor skills, one occupational therapist with at least 3 years’ experience in early childhood development within community settings, and one teacher with a minimum of 3 years’ experience in preschool education. Then, the indexes of item–objective congruence (IOC) were calculated. The results of the IOC and suggestions from experts were discussed in order to improve the activities of each age group. In Phase 2, the classroom teachers were trained in how to implement the intervention program in order to foster the development of fine motor skills. Each teacher received a set of activities designed to promote fine motor skill development, aligned with the age ranges of their students. This set comprised 20 activity sessions, each lasting 30 min, in total 10 h. Each session was structured into two 15‐min segments that consisted of bilateral motor coordination and visual–motor integration activities. Then, feasibility and acceptability were examined as follows.

#### 2.2.1. Feasibility

Feasibility of the program was examined by using the DSPM screening tool to indicate developmental progression of the preschoolers, which in routine practice produces a categorical classification by a binary outcome, “suspected” versus “age‐appropriate/normal.” McNemar’s test was employed for paired dichotomous data to evaluate the feasibility of the intervention by assessing the significance of the change in fine motor development (“still delayed” vs. “normal/improved”) of the same group of children from pre‐ to postintervention. This test served as a practical and efficient tool for early‐phase feasibility assessments. In this study, the participants were assessed for fine motor development before the intervention and then re‐evaluated after it. The outcomes were dichotomized into two categories: (1) “normal (improved)” and (2) “still delayed (no change).” Dichotomization with binary outcomes in pilot feasibility helped to establish whether the program warranted further investigation through larger scale efficacy or implementation studies. In addition, with *n* = 30, cell counts in more granular categories would be small and unstable. Dichotomization reduces sparsity and avoids violated assumptions or unreliable estimates that can occur with many small subcategories.

#### 2.2.2. Acceptability

**Figure 1:**
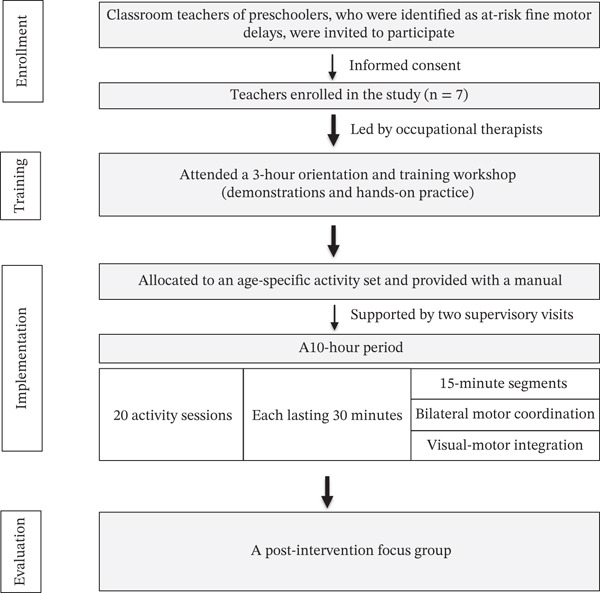
The process of teacher preparation and implementation.

The acceptability in this study was defined as satisfaction of teachers, who used the intervention program in preschool students suspected of delayed fine motor development by the DSPM. After implementation, seven teachers were asked to take part in a focus group to discuss and provide feedback regarding the intervention program. An interview guide was used to conduct the focus group and ask questions pertaining to acceptability of the intervention, as well as including changes in fine motor skills observed by the students. The focus group was recorded and transcribed verbatim. Qualitative data transcripts were read repeatedly to achieve immersion, followed by open coding to identify meaningful segments reflecting program usability, engagement, and observed child outcomes. The codes were categorized and refined through axial coding to highlight relationships among emerging concepts. To enhance reliability, two independent researchers conducted parallel coding, resolving discrepancies through discussion until consensus was reached. Finally, thematic analysis was conducted to identify key themes, and peer debriefing with a senior researcher was employed to validate the thematic structure. The CONSORT diagram used to illustrate the process of teacher preparation and implementation within the pilot intervention program is presented in Figure 1.

## 3. Results

### 3.1. Intervention Program to Promote Fine Motor Skills in Preschoolers

The final intervention program comprised 97 activities, including 33 activities of bilateral motor coordination and 64 of visual motor integration. Thirty‐three bilateral motor coordination activities comprised 14 activities of symmetrical bilateral motor coordination, eight of asymmetrical bilateral motor coordination, and 11 of dominant and stabilizing sides. Sixty‐four visual motor integration activities comprised 17 activities of construction play, eight of completing puzzles, four of tracing lines, four of copying lines, two of tracing shapes, eight of copying shapes, three of drawing complex lines, two of tracing letters, two of tracing numbers, two of making numbers, four of drawing figures, six of copying letters, and two of copying numbers. In addition, these activities were considered in each age group and contained gradually in 20 activity periods. The intervention program consisted of 20 activity periods matched to each child’s age group, which are presented in Table 2.

**Table 2:** Intervention program to promote fine motor skills in each age group.

Age group	Activity period	Implementation activity	
		BMC/manipulation activity	VMI activity
I	1	Symmetrical BMC: Level 1	Construction play: Level 1
	2	Symmetrical BMC: Level 2	Construction play: Level 2
	3	Symmetrical BMC: Level 3	Construction play: Level 3
	4	Symmetrical BMC: Level 4	Construction play: Level 4
	5	Symmetrical BMC: Level 5	Construction play: Level 5
	6	Symmetrical BMC: Level 6	Construction play: Level 6
	7	Symmetrical BMC: Level 7	Construction play: Level 7
	8	Symmetrical BMC: Level 8	Construction play: Level 8
	9	Symmetrical BMC: Level 9	Completing puzzles: Level 1
	10	Symmetrical BMC: Level 10	Completing puzzles: Level 2
	11	Symmetrical BMC: Level 11	Completing puzzles: Level 3
	12	Symmetrical BMC: Level 12	Completing puzzles: Level 4
	13	Alternating BMC: Level 1	Tracing lines: Level 1
	14	Alternating BMC: Level 2	Tracing lines: Level 2
	15	Alternating BMC: Level 3	Tracing lines: Level 3
	16	Alternating BMC: Level 4	Tracing lines: Level 4
	17	Dominant and stabilizing sides together: Level 1	Copying lines: Level 1
	18	Dominant and stabilizing sides together: Level 2	Copying lines: Level 2
	19	Dominant and stabilizing sides together: Level 3	Copying lines: Level 3
	20	Dominant and stabilizing sides together: Level 4	Copying lines: Level 4

II	1	Symmetrical BMC: Level 3	Construction play: Level 3
	2	Symmetrical BMC: Level 4	Construction play: Level 4
	3	Symmetrical BMC: Level 5	Construction play: Level 5
	4	Symmetrical BMC: Level 6	Construction play: Level 6
	5	Symmetrical BMC: Level 7	Construction play: Level 7
	6	Symmetrical BMC: Level 8	Construction play: Level 8
	7	Symmetrical BMC: Level 9	Construction play: Level 9
	8	Symmetrical BMC: Level 10	Construction play: Level 10
	9	Symmetrical BMC: Level 11	Completing puzzles: Level 1
	10	Symmetrical BMC: Level 12	Completing puzzles: Level 2
	11	Alternating BMC: Level 1	Completing puzzles: Level 3
	12	Alternating BMC: Level 2	Completing puzzles: Level 4
	13	Alternating BMC: Level 3	Tracing lines: Level 3
	14	Alternating BMC: Level 4	Tracing lines: Level 4
	15	Alternating BMC: Level 5	Tracing shapes: Level 1
	16	Dominant and stabilizing sides together: Level 1	Tracing shapes: Level 2
	17	Dominant and stabilizing sides together: Level 2	Copying lines: Level 3
	18	Dominant and stabilizing sides together: Level 3	Copying lines: Level 4
	19	Dominant and stabilizing sides together: Level 4	Copying shapes: Level 1
	20	Dominant and stabilizing sides together: Level 5	Copying shapes: Level 2

III	1	Symmetrical BMC: Level 5	Construction play: Level 7
	2	Symmetrical BMC: Level 6	Construction play: Level 8
	3	Symmetrical BMC: Level 7	Construction play: Level 9
	4	Symmetrical BMC: Level 8	Construction play: Level 10
	5	Symmetrical BMC: Level 9	Construction play: Level 11
	6	Symmetrical BMC: Level 10	Construction play: Level 12
	7	Symmetrical BMC: Level 11	Completing puzzles: Level 2
	8	Symmetrical BMC: Level 12	Completing puzzles: Level 3
	9	Alternating BMC: Level 1	Completing puzzles: Level 4
	10	Alternating BMC: Level 2	Completing puzzles: Level 5
	11	Alternating BMC: Level 3	Tracing shapes: Level 1
	12	Alternating BMC: Level 4	Tracing shapes: Level 2
	13	Alternating BMC: Level 5	Copying lines: Level 3
	14	Alternating BMC: Level 6	Copying lines: Level 4
	15	Dominant and stabilizing sides together: Level 1	Copying shapes: Level 1
	16	Dominant and stabilizing sides together: Level 2	Copying shapes: Level 2
	17	Dominant and stabilizing sides together: Level 3	Copying shapes: Level 3
	18	Dominant and stabilizing sides together: Level 4	Copying shapes: Level 4
	19	Dominant and stabilizing sides together: Level 5	Copying shapes: Level 5
	20	Dominant and stabilizing sides together: Level 6	Copying shapes: Level 6

IV	1	Symmetrical BMC: Level 8	Construction play: Level 13
	2	Symmetrical BMC: Level 9	Construction play: Level 14
	3	Symmetrical BMC: Level 10	Construction play: Level 15
	4	Symmetrical BMC: Level 11	Completing puzzles: Level 4
	5	Symmetrical BMC: Level 12	Completing puzzles: Level 5
	6	Symmetrical BMC: Level 13	Completing puzzles: Level 6
	7	Alternating BMC: Level 1	Copying shapes: Level 4
	8	Alternating BMC: Level 2	Copying shapes: Level 5
	9	Alternating BMC: Level 3	Copying shapes: Level 6
	10	Alternating BMC: Level 4	Copying shapes: Level 7
	11	Alternating BMC: Level 5	Drawing complex lines: Level 1
	12	Alternating BMC: Level 6	Drawing complex lines: Level 2
	13	Alternating BMC: Level 7	Tracing letters: Level 1
	14	Dominant and stabilizing sides together: Level 4	Tracing letters: Level 2
	15	Dominant and stabilizing sides together: Level 5	Copying letters: Level 1
	16	Dominant and stabilizing sides together: Level 6	Copying letters: Level 2
	17	Dominant and stabilizing sides together: Level 7	Tracing numbers: Level 1
	18	Dominant and stabilizing sides together: Level 8	Tracing numbers: Level 2
	19	Dominant and stabilizing sides together: Level 9	Making numbers: Level 1
	20	Dominant and stabilizing sides together: Level 10	Making numbers: Level 2

V	1	Symmetrical BMC: Level 11	Construction play: Level 16
	2	Symmetrical BMC: Level 12	Construction play: Level 17
	3	Symmetrical BMC: Level 13	Completing puzzles: Level 7
	4	Symmetrical BMC: Level 14	Completing puzzles: Level 8
	5	Alternating BMC: Level 1	Copying shapes: Level 7
	6	Alternating BMC: Level 2	Copying shapes: Level 8
	7	Alternating BMC: Level 3	Drawing complex lines: Level 2
	8	Alternating BMC: Level 4	Drawing complex lines: Level 3
	9	Alternating BMC: Level 5	Drawing figures: Level 1
	10	Alternating BMC: Level 6	Drawing figures: Level 2
	11	Alternating BMC: Level 7	Drawing figures: Level 3
	12	Alternating BMC: Level 8	Drawing figures: Level 4
	13	Dominant and stabilizing sides together: Level 4	Copying letters: Level 1
	14	Dominant and stabilizing sides together: Level 5	Copying letters: Level 2
	15	Dominant and stabilizing sides together: Level 6	Copying letters: Level 3
	16	Dominant and stabilizing sides together: Level 7	Copying letters: Level 4
	17	Dominant and stabilizing sides together: Level 8	Copying letters: Level 5
	18	Dominant and stabilizing sides together: Level 9	Copying letters: Level 6
	19	Dominant and stabilizing sides together: Level 10	Copying numbers: Level 1
	20	Dominant and stabilizing sides together: Level 11	Copying numbers: Level 2

### 3.2. Feasibility of the Intervention Program to Promote Fine Motor Skills in Preschoolers

The findings of this study presented feasibility in implementing the intervention program to promote fine motor developmental progression among preschoolers across various age groups. A total of 22 children (73.33%) exhibited improvement in fine motor development, thus achieving age‐appropriated developmental status postintervention. In contrast, eight children (26.67%) did not demonstrate significant changes in their fine motor skills. Notably, the greatest improvement was observed in Age Group III, in which all of the children showed developmental progression and none remained unchanged. McNemar’s test was employed to assess the significance of these changes statistically. The statistically significant result provided robust evidence in that the intervention contributed to the observed developmental progress (*χ* ^2^ = 5.63, df = 1, *p* ~ 0.001). The odds ratio (OR) of improvement was 2.75 with 95% confidence interval (CI) (95% CI: 1.22–6.18), which indicated significant effect of the intervention on improving fine motor development among the participants. In addition, the results revealed that most participants achieved improvements, and no harmful effects were reported. Results of fine motor developmental progression after implementation are presented in Table 3.

**Table 3:** Results of fine motor developmental progression after implementation.

Age group	Numbers (percentage)		OR	*χ* ^2^	*p* value
	Normal (improved)	Still delayed (no change)			
I	3 (10.00)	2 (6.67)			
II	4 (13.33)	2 (6.67)			
III	7 (23.33)	0 (0.00)			
IV	3 (10.00)	3 (10.00)			
V	5 (16.67)	1 (3.33)			
Total	22 (73.33)	8 (26.67)	2.75	5.63	0.000 ^∗^

^*^ *p* < 0.001.

### 3.3. Acceptability of the Intervention Program to Promote Fine Motor Skills in Preschoolers

The three key themes regarding acceptability of the intervention program included engagement and appropriateness of the materials, effectiveness in promoting fine motor and academic skills, and practicality and usability for teachers. The summary of key themes is presented in Table 4.

**Table 4:** Summary of key themes.

Theme	Description
1. Engagement and appropriateness of materials	The toys and materials used in the intervention were colorful, and they motivated interest and engagement in activities. While the majority of materials were suitable in weight and design, some such as crayons and worksheet images may require adjustments in size for better usability.
2. Effectiveness in promoting fine motor and academic skills	The intervention effectively supported the development of fine motor skills and enhanced readiness for academic tasks. Its activities were beneficial for both children typically developing and those with delays.
3. Practicality and usability for teachers	The program was user‐friendly, with clear manuals and easy‐to‐follow instructions. It helped to reduce duration of planning, support differentiated instruction, and provide flexible tools that teachers can adapt to regarding the students’ performance levels.

Details of each key theme are as follows.

#### 3.3.1. Theme1: Engagement and Appropriateness of Materials

The materials in each activity were appreciated by the teachers. Their colorful, engaging, and attractive features helped to capture the children’s interest and encourage participation. The students were naturally drawn to the toys and excited to engage in activities with them, indicating success in motivation through play‐based learning. However, there were some concerns regarding the size of certain materials, such as crayons and worksheet images. These issues affected accessibility for younger children or children with smaller hands. Nevertheless, the teachers also highlighted the weight of the toys that was generally well‐suited for preschoolers, providing good sensory experience without being too heavy to handle. Despite some materials being either too large or too small, the overall presentation and sensory appeal were considered strengths of the program. Illustrative examples of statements are as follows:

• *“Some toys are quite big for the students’ hands.”—Teacher 1*
• *“The students find the toys interesting and [they] want to play. It motivates them.”—Teacher 3*
• *“The crayon is quite small. If it were changed to be larger, it would be suitable for the children’s hand size.”—Teacher 4*
• *“The toys are colorful and safe. The size is suitable for the hands of preschoolers.”—Teacher 7.*

#### 3.3.2. Theme 2: Effectiveness in Promoting Fine Motor and Academic Skills

The teachers consistently emphasized the positive impact of intervention on the children’s fine motor development. The activities supported crucial skills such as grasping, pinching, and manipulating small objects, and prewriting readiness. Moreover, teachers noted that the activities not only facilitated developmental progression but also developed academic interest and encouraged educational performance, particularly in writing and drawing tasks. Some activities were initially seen as challenging, especially for younger or slow‐developed children, but with repetition and familiarization, the students improved their skills significantly. The program was also considered beneficial for children with delayed fine motor development, indicating its inclusivity and adaptability to various developmental levels. Illustrative examples of statements are as follows:

• *“Some activities might be challenging for the students at first, but once they become familiar, they can learn and improve their fine motor skills.”—Teacher 1*
• *“The activities not only promote fine motor skills but also encourage academic skills, especially in motivating the students to engage in writing activities easily.”—Teacher 2*
• *“The program is useful for promoting fine motor skills in children with delayed fine motor skills.”—Teacher 4*
• *“It benefits are promoting fine motor skills, preparing pre-handwriting readiness, and providing a background for academic performances.”—Teacher 6.*

#### 3.3.3. Theme 3: Practicality and Usability for Teachers

One of the standout features of the program, as mentioned by all of the teachers, is its user‐friendly design and practicality for classroom use. The manual was praised for its clarity, visual guidance, and step‐by‐step instructions, making it easy for teachers to implement the activities without requiring extensive preparation. This was particularly appreciated by teachers managing classrooms with diverse developmental needs. The intervention was viewed as a time‐saving tool, especially useful in reducing the effort needed to plan targeted fine motor activities specifically for students with suspected delayed fine motor development. Additionally, the program was seen as a resource for teachers to develop or adapt their own classroom activities. However, some teachers suggested that it would be more effective to categorize student activities by performance or class level than by age group. They opined that the students had varying abilities even within the same age group. Illustrative examples of statements are as follows:

• *“The manual is very useful for following the activities and easy to understand.”—Teacher 1*
• *“Although they are the same age, their performance is different, so if the set of activities is categorized by performance, it will be better.”—Teacher 2*
• *“This program is easy to pick up and use with students, decreasing the time needed for activity planning for teachers who need to promote development.”—Teacher 4*
• *“The activity can bring ideas to adapt and create learning activities in the classroom.”—Teacher 6.*

Program adherence was monitored through teacher logs and supervisory visits. All seven teachers completed the 20 activity sessions within the 10‐h period by following the age‐specific manuals provided. Supervisory visits by occupational therapists confirmed that activities were implemented according to the structured dual‐component format: bilateral motor coordination and visual–motor integration. Minor adjustments were made to accommodate classroom routines, but all of the teachers were able to follow the intervention protocol.

## 4. Discussion

This pilot study provides initial evidence of the feasibility and acceptability of an early intervention program aimed at enhancing fine motor skills in preschoolers. In terms of feasibility, the majority of participants presented improvement in each age group. The consistent positive progression suggests that the intervention can be promising preliminary evidence in various age groups. This finding aligns with the systematic review and meta‐analysis by Strooband et al. [15], which examined motor skill interventions to improve fine motor development in children aged birth to 6 years. Their review reported positive intervention effects and moderate effect sizes for motor skill programs on fine motor, visual–motor, and manual dexterity outcomes. Moreover, in this pilot study, findings revealed that the children experienced no injury, fatigue, or regression in fine motor skills. However, eight out of 30 children (26.67%) showed no improvement in fine motor development after the intervention. This can be considered an adverse or nonresponsive outcome, suggesting that the program may not benefit all children equally. In particular, the results indicated that all participants who still presented delayed development were at the younger age of each age group. Regarding the structural design, the activities implemented a comprehensive approach to fine motor skill development by integrating the crucial fundamental components, particularly bilateral motor coordination and visual–motor integration [16–19]. These activities were designed with a play‐based approach that involved manipulating small objects, drawing, and using tools, which were relevant to the occupations of preschoolers [20]. Therefore, structured and age‐appropriate occupation programs could have preliminary findings that enhance fine motor proficiency. This approach supports children’s learning experiences and provides essential resources for educational success. The findings are valuable for occupational therapists, related professionals, and policymakers in designing targeted interventions. Furthermore, empowering teachers with knowledge and resources fosters collaborative practices with occupational therapists, enabling the delivery of early intervention services and the promotion of fine motor skills in community preschool settings. This relates to areas of occupational therapy that focus on evaluating all aspects of the domain: their interrelationships and impact on the client when participating in daily life occupations [3].

The practical implications of these findings emphasize not only feasibility but also acceptability of the users. Beyond improvements in fine motor development among most participants, teachers provided positive feedback regarding the program’s engagement, appropriateness of materials, effectiveness in promoting fine motor and preacademic skills, and practicality for classroom use indicated acceptability [21]. In terms of appropriateness of the materials, the colorful and attractive nature of the toys and materials effectively captured the children’s interest, due to their design alongside age‐appropriated play materials. Therefore, this could support the principles of play‐based early learning in preschoolers [22]. In addition, the teachers observed the program’s effectiveness in promoting fine motor skills as well as preacademic abilities, particularly prewriting readiness. The program’s potential could be a time‐saving tool for planning targeted fine motor activities and a source for developing or adapting classroom activities for teachers in a community. Well‐developed fine motor skills underpin academic tasks such as writing and drawing and positively influence school readiness [4, 5]. Moreover, a clear manual with visual guidance and user‐friendly step‐by‐step instructions could support the implementation of an intervention program for teachers [23]. For these reasons, the program’s practicality and usability were key factors contributing to its acceptability.

However, the teachers suggested categorizing activities by performance level rather than strict age group. This highlighted the importance of differentiating instructions of the intervention program concerning the preschoolers’ current performance as well as their chronological age. This aligns with pedagogical best practices that recognize the variability in developmental trajectories among children of the same age [24]. In addition, the concerns raised about the size of some materials (crayons and worksheet images) emphasize the importance of accommodating younger children or those with smaller hands. Addressing these practical issues is essential for optimizing the program’s usability. Moreover, ease of administration, minimal resistance to participation, and observable short‐term benefits support the program’s broader application in educational and clinical contexts.

Furthermore, this community early intervention program could be a collaborative tool between education professionals and occupational therapists in a community setting that lacks occupational therapists. While pediatric occupational therapists typically work in multidisciplinary teams with teachers in schools [25], limited community positions and broad caseloads across age groups [13, 25] pose challenges in delivering services to at‐risk preschoolers in child development centers. This might decrease the opportunity for preschoolers to participate in their age‐appropriated occupations. In the perspective of occupational therapy, teachers are the crucial environmental factor for preschoolers. When teachers are confident and free from worry, the preschoolers learn with positive support and relationships in a community school setting that conducts the students’ functioning and daily occupations [3]. Therefore, the occupational therapists in this study designed teacher‐led activities in the intervention program, with suggestions from related experts to promote fine motor skills for preschoolers. Integrating these activities into natural classroom routines may offer teachers a comfortable means of supporting fine motor development.

### 4.1. Limitations and Future Research

Clinically, the program could be integrated into community child development centers to support children who are at risk of developmental delays. Structured activities targeting bilateral motor coordination and visual‐motor integration can enhance fine motor skills, which are essential for daily functioning and academic readiness. Despite the promising results, this study has several limitations. Firstly, it was a pilot investigation with a small sample of 30 preschoolers and seven teachers, limiting generalizability. Additionally, the study was conducted in a specific community child development center in Chiang Mai province, Thailand, which may not reflect the diversity of preschool environments and populations elsewhere. Furthermore, reliance on the DSPM screening tool may not capture all aspects of fine motor proficiency. Lastly, the intervention program’s effectiveness was assessed over a relatively short period, and long‐term impacts on fine motor skills and overall development remain uncertain.

Future studies should consider comprising larger sized and more diverse samples and include multiple measures of motor skill development to validate and extend these findings. Moreover, employing randomized controlled trial designs would enhance the rigor of the research and minimize potential biases. Additionally, incorporating multiple measures of fine motor skills, beyond the DSPM, would provide a more comprehensive assessment of children’s fine motor proficiency. Long‐term studies are essential to evaluate the sustained impact of intervention programs on fine motor skills and overall development, as well as assess the cost‐effectiveness of the program, which is crucial in determining its practicality for widespread adoption. Furthermore, an explorative study regarding the integration of health technology could further enhance effectiveness and accessibility. Finally, studies should explore scalability across diverse communities and educational settings to assess whether the intervention can be sustainably implemented on a larger scale. In particular, the study of acceptability might expand to broader users and measures of usability testing for evaluating long‐term impact from the user’s perspective.

## 5. Conclusion

This pilot quasi‐experimental study gave promising preliminary evidence of the feasibility and acceptability of an intervention program designed to promote fine motor skill development in preschoolers with fine motor developmental delay. Feasibility of the intervention program was indicated by statistically significant postintervention improvement in the fine motor skills of preschoolers, particularly within the 4.0–4.11‐year age group (Group III). Acceptability was revealed by positive feedback from teachers as potential users. This feedback highlighted engagement of the program’s materials, positive impact on both fine motor and academic skills, and practicality within the classroom setting. The positive outcomes in terms of the children’s development and teachers’ perceptions suggest that this collaborative approach between educators and occupational therapists holds potential value for promoting early childhood development, and addressing the challenges posed by limited access to occupational therapy services in community settings. However, this study had a small sample size and lack of a control group. Future studies with a larger sample size and controlled designs are warranted to evaluate the efficacy and long‐term impact of this intervention more rigorously. In addition, the program’s structured, teacher‐led design suggests potential scalability. With appropriate adaptation, the program could be scaled to other community child development centers or integrated into broader early childhood education systems, thereby strengthening its practice relevance.

## Author Contributions

**Suchitporn Lersilp**: reviewed the literature, designed the research, collected and analyzed the data, and developed the manuscript. **Kewalin Panyo**: collected and analyzed the data, shared opinions on the data analysis and discussion, and approved the manuscript as the essential intellectual contributor. **Napalai Chaimaha**: collected and analyzed the data, shared opinions on the findings, approved the manuscript, and served as the corresponding author. **Supawadee Putthinoi**: provided advice on the research design and approved the manuscript. **Autchariya Punyakaew**: collected the research and shared opinions on the findings. **Sasithorn Sung-U**: shared opinions on designing the research.

## Funding

This work was supported by the matching fund from the Department of Occupational Therapy and Faculty of Associated Medical Sciences, Chiang Mai University, Thailand.

## Ethics Statement

This study was approved by the Research Ethics Committee of the Faculty of Associated Medical Sciences, Chiang Mai University, Thailand (AMSEC‐66EX‐116).

## Consent

All of the participants provided informed consent.

## Conflicts of Interest

The authors declare no conflicts of interest.

## Data Availability

The data that support the findings of this study are available from the corresponding author on reasonable request.
